# A Collision Probability Model of Portal Vein Tumor Thrombus Formation in Hepatocellular Carcinoma

**DOI:** 10.1371/journal.pone.0130366

**Published:** 2015-07-01

**Authors:** Fei Xiong

**Affiliations:** Department of Gastroenterology,Sichuan Academy of Medical Sciences &Sichuan Provincial People’s Hospital, Chengdu, Sichuan, PR China; The University of Hong Kong, CHINA

## Abstract

Hepatocellular carcinoma is one of the most common malignancies worldwide, with a high risk of portal vein tumor thrombus (PVTT). Some promising results have been achieved for venous metastases of hepatocellular carcinoma; however, the etiology of PVTT is largely unknown, and it is unclear why the incidence of PVTT is not proportional to its distance from the carcinoma. We attempted to address this issue using physical concepts and mathematical tools. Finally, we discuss the relationship between the probability of a collision event and the microenvironment of the PVTT. Our formulae suggest that the collision probability can alter the tumor microenvironment by increasing the number of tumor cells.

## Introduction

Hepatocellular carcinoma(HCC) is the sixth most common malignancy and the third most frequent cause of cancer death [1]worldwide. When locally advanced HCC invades the intrahepatic vasculature, its metastatic cells tend to invade the portal vein system with tumor emboli, a phenomenon known as portal vein tumor thrombus (PVTT). The incidence of portal vein tumor thrombus is 44%–62.8% according to autopsy results [2, 3] and 31.4%–34% according to clinical data [4, 5]. PVTT indicates a poor prognosis for patients with hepatocellular carcinoma (HCC); however, the mechanism of PVTT formation is still unknown. Obviously, the mechanism underlying the invasion and metastasis of tumors plays an important role in PVTT development. The theoretical foundation of this mechanism is the “epithelial-mesenchymal transition”(EMT), a process by which transformed epithelial cells gain the ability to invade, metastasize [6] and broadly regulate further invasion and metastasis. However, the theory does not explain why primary tumor cells can colonize certain distant tissue sites after they enter into circulation [7].

This problem has been approached from another point of view: the “tumor microenvironment” [6]. For example, Yang P et al. reported a correlation between hepatitis B virus (HBV) infection and the formation of PVTT [8]. The researchers provided experimental support for their theory: TGF-beta and cytokines/chemokines create a favorable microenvironment for PVTT formation through the regulation of Treg cell recruitment. This finding represents a step forward, but a key question still remains: why is the incidence of PVTT not dependent on the proximity to its primary origin after hepatic metastases enter the blood-flow pathway [9]. The tumor microenvironment may be only part of the explanation, as it does not explain why the PVTT has a particular microenvironment. It is possible that the tumor microenvironment or cell signaling and gene regulation cannot explain the formation of PVTT; thus, the problem may need to be studied from a different angle.

## Theory

### Principle of least action

In physics, it is known that the principle of least action is very important. Because the principle is so universal in nature, it provides the basis of our PVTT model. By treating the neoplastic cells as particles in a vein, their motion can be described by the particles’velocities and positions. Thus, the least-action principle is represented by a Lagrangian function, as follows [10]:

$$S=\int_{t_{1}}^{t_{2}}\zeta \;(X_{i}:V_{i})dx$$ (1)

This function describes a quantity called the action, S, which is defined as the integral of the Lagrangian *ζ* between two time points *t*_1_and *t*_2_. In addition, *X*_*i*_ and *V*_*i*_ are the position and velocities of the particles. According to the calculus of variations [10],

δS=0 (2)

where *δ* indicates a small change between the minimum and a slight level of motion.

According to this differential statement, the least-action principle corresponds to a particle that senses all of the paths in the neighborhood and chooses the one with the least action [10]. Therefore, the principle of least action provides a logical base for our PVTT model. In other words, by modeling cancer cells that have escaped from the primary HCC site into the portal venous system as particles, we attempt to show that these particles have the least action in the portal venous branches.

### Establishment of rectangular coordinates in a three-dimensional space

The motion of a body can only be described relative to something else. We call this “something else” the frame of reference [11], which may be determined by other bodies, observers, or a set of time-space coordinates. If the frames of reference is chosen badly, the laws of motion maybe more complex than necessary. Thus, in describing the tumor particle motion in the PVTT model, it is important to choose an appropriate reference frame.

Most motions in a 3D environment are modeled using a rectangular coordinate system. In our model, we found that it was simplest to adhere to the Cartesian coordinate system.

However, some details remain to be determined in the coordinate system. The tumor particle’s motion should be expressed in terms of the X, Y, and Z components. Thus, the motion can be written as Eq (11)

$$\Delta f\;(x,y,z,)=\frac{\partial f}{\partial x}\Delta x+\frac{\partial f}{\partial y}\Delta y+\frac{\partial f}{\partial z}\Delta z$$ (3)

Let us suppose that the rectangular coordinate system forms a set of 3 components. We must then reduce the number of variables and decrease the computational complexity. In our case, we assume that the direction of total energy is parallel to the z component.

The next chapter describes the field of view in the portal vein system.

### Characteristics of the vector field in a portal vein system

The portal vein is a liver blood vessel that moves blood from the digestive tract, spleen, pancreas and gallbladder to the liver [12]. In short, the portal vein flow follows a “toward liver” pattern. In most cases, HCC develops with an established background of chronic liver disease [13], and malignant hepatic cell transformation is more frequent in cirrhotic livers, accounting for 80%–90% in a previously reported autopsied series [14]. The cirrhosis results indicate hemodynamic change and portal hypertension. Thus, it is no surprise that a hepatofugal flow is relatively common in patients with cirrhosis [15]. Therefore, it is safe to assume that the hepatofugal flow is the general direction of external force in the PVTT model.

In our model, we ignore the viscous effects and the wall flexibility in vein(For our model, we should follow the basic law of simplification. these factors are covered detailedly in the result part and discussion part). For a liquid with no viscosity, only the inertial force and pressure are considered. According to Newton’s second law and the definition of pressure [16], we write:

$$f_{m}=M_{tumor}av_{tumor}+\Delta Ps_{tumor}$$ (4)

where *f*_*m*_ is the force on the tumor particle, Δ*P* is the pressure on the tumor particle, *a* is the acceleration, *v*_*tumor*_ is the volume of the particle, *s*_*tumor*_ is the contact area of the surface.

We now describe our PVTT model from a different viewpoint. We subdivide the length of all of the blood vessels into subintervals in the portal vein system and let the norm of the vein partitions approach zero. In other words, the blood vessels are sliced into cylinders, and the length of each cylinder approaches zero.

**Fig 1:**
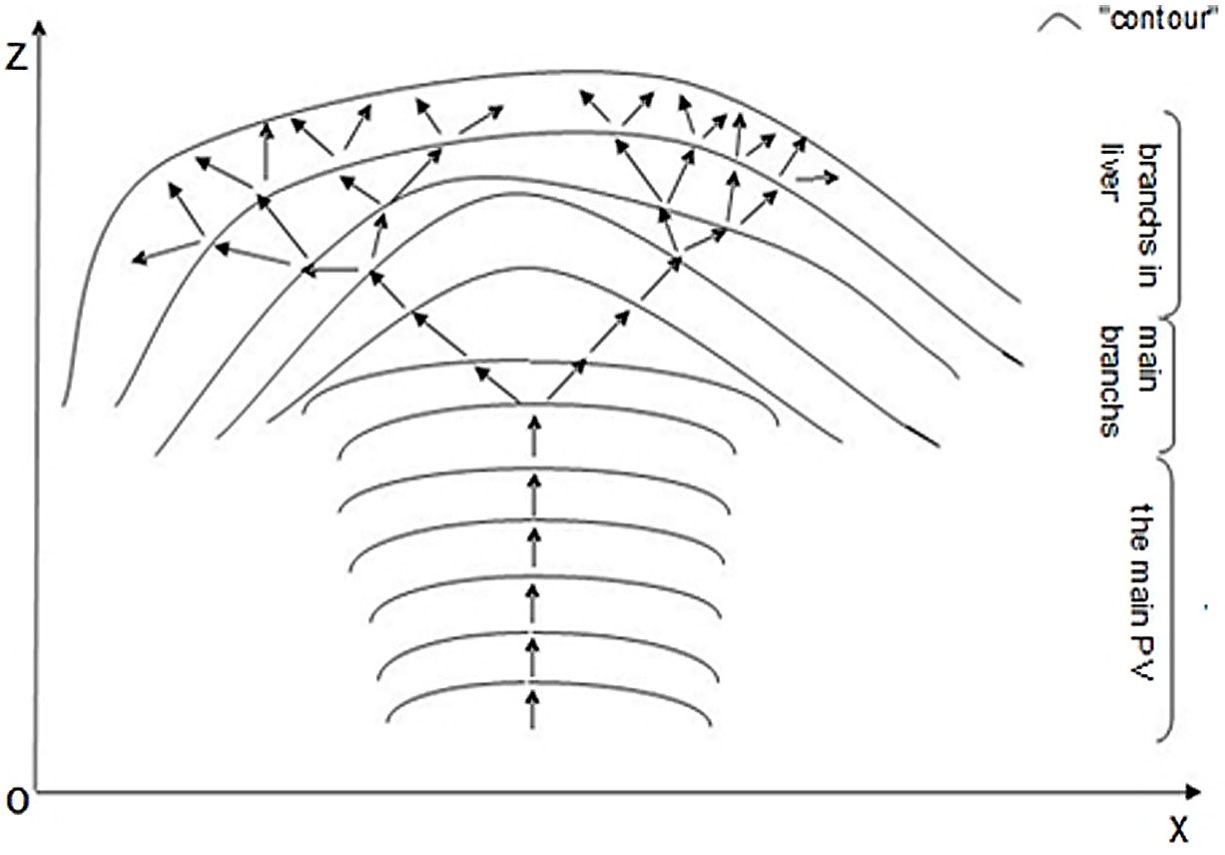
An image of the vector field of a portal vein. The arrows represent the various energy transfer pathways. The curve represents a “contour” that passes through all of the arrows at the same time, where time is projected onto the model as the fourth dimension.

According to the conservation of energy, there are several different energy transfer pathways in the portal vein system because the vein distributions vary from one to another. These pathways are defined as arrows. Let us imagine “contour”corresponding to imaginary surfaces drawn through all points at which the fields have the same moment(cylinder is approximated as point on contour curve). Thus, we will use these arrows and “contours”in our description of energy from a vector viewpoint, which will aid in illustrating the physics of the portal vein tumor thrombus model. In addition, time (the 4th dimension) is projected as a “contour”of the model. The general vector field of the portal vein system is shown (Fig 1).

The general vector field (Fig 1) corresponds to a normal hemodynamic state. When the general flow in the PVTT model follows a hepatofugal flow, the arrows point in the opposite direction. At this point, we incorporate two facts into our model. 1. For the portal vein system(portal vein system is approximately considered as an isolate system), the total energy of a steadily moving fluid remains constant over time. Thus, its energy is equivalent at different time points. 2. According to 1, the general force is the vector sum of the forces from a“contour” in the vein system. These constraints are based on the“conservation of energy”[10] and“the principle of superposition”[10]. Eq (5) states that

$$E_{sum}=t\sum_{k=e}^{n}e$$ (5)

where *E*_*sum*_ is the sum of energy in the cylinders relative to the entire portal vein vector field, t is a “contour” at a given time, n is the number of arrows at a “contour”, k is a summation index beginning at e, and e is a random energy for a given “contour”.

In the distribution of portal veins, one may notice an interesting trend in its vector field (Fig 1). The arrows for the “contours” in the main portal vein should be parallel. According to Eq (5), the direction of *E*_*sum*_ tends to be parallel to the major branch; thus, it is logical to use the direction of the main portal vein as the z axis.

### Directional Derivatives in PVTT’s model

This method describes the movement of tumor particles when a hepatofugal flow is dominant in the model. In other words, the arrows (Fig 1) point in the opposite direction.

Suppose that a tumor particle corresponds to a point in the PVTT model, such that *p*_0_(*x*_0_, *y*_0_, *z*_0_) is the location of the tumor particle and *u* = *u*_1_ *i*+*u*_2_ *j*+*u*_3_ *k* is a unit vector for a circular cross-section of the cylinder. Then, we obtain Eq (6):

$$\begin{array}{c} x=x_{0}+su_{1}\\ y=y_{0}+su_{2}\\ z=z_{0}+su_{3} \end{array}$$ (6)

where s is the length of an arc from *p*_0_ in the direction of *u*.

According to Eq (4) and the tumor particle’s directional derivatives, *f*(*x*_0_, *y*_0_, *z*_0_) is a constant related to the length of the cylinder at the point *p*_0_(*x*_0_, *y*_0_, *z*_0_) and (*D*_*u*_ *f*)_*p*_0__ is the instantaneous rate of change of this value at *p*_0_ step in the direction of *u*[16], compare to *f*(*x*, *y*, *z*). We then find:

$$\begin{array}{cc} {\left(D_{u}f\right)\;}_{p_{0}} & =\left(\frac{\partial f}{\partial x}\right)\;p_{0}\frac{dx}{ds}+\left(\frac{\partial f}{\partial y}\right)\;p_{0}\frac{dy}{ds}+\left(\frac{\partial f}{\partial z}\right)\;p_{0}\frac{dz}{ds}\\ & =\left(\frac{\partial f}{\partial x}\right)\;p_{0}.u_{1}+\left(\frac{\partial f}{\partial y}\right)\;p_{0}.u_{2}+\left(\frac{\partial f}{\partial z}\right)\;p_{0}.u_{3}\\ & =\left[\left(\frac{\partial f}{\partial x}\right)\;p_{0}i+\left(\frac{\partial f}{\partial y}\right)\;p_{0}j+\left(\frac{\partial f}{\partial z}\right)\;p_{0}k\right].\left(u_{1}i+u_{2}j+u_{3}k\right) \end{array}$$ (7)

The equation of motion for a tumor particle is established by this formula Eq (7), which decomposes the path into two parts: 1. The first part of the formula gives the rate of change of *f*with respect to the length of the cylinder when movement is ingored in the circular cross-section. 2. The last part of the formula is similar to the first part, but considers the circular cross-section of the cylinder. Namely, (*D*_*u*_ *f*)_*p*_0__ is the dot product of the two types of motion for the tumor particle.

## Results

We showed in chapter 2.3 that our model is composed of a series of cylinders. As the length of the cylinder approaches zero, we must supplement our model with a numerical technique: we use a circular cross-section in place of a cylinder when the length approaches zero. This method is called “Newton’s method” [17].

First, we characterize the tumor particle motion with respect to the circular cross-section of the cylinder using the following function:

r=g(t)+h(t)+k(t) (8)

where *t* represents the 4th dimension of time. We find that the equation gives a smooth curve of motion in the circular cross-section. We now aim to visualize the behavior of a tumor particle. Let M be the plane of the circular cross-section, which we assume has a constant value(the constant value represents a point in 3-D space, it can also be seen as a real-value of a function of three variables:x, y, z). If the particle instantaneously moves in the M plane in any direction, then we have *f*(*g*(*t*), *h*(*t*), *k*(*t*)); by differentiating both sides of this equation with respect to t, we obtain the following formula:

$$\begin{array}{ccc} \frac{d}{dt}f\;(g\;(t),h\;(t),k\;(t)) & = & \frac{d}{dt}\;(c)\\ \frac{\partial f}{\partial x}\frac{dg}{dt}+\frac{\partial f}{\partial y}\frac{dh}{dt}+\frac{\partial f}{\partial z}\frac{dk}{dt} & = & 0\\ \left(\frac{\partial f}{\partial x}i+\frac{\partial f}{\partial y}j+\frac{\partial f}{\partial z}k).(\frac{dg}{dt}i+\frac{dh}{dt}j+\frac{dk}{dt}k\right) & = & 0 \end{array}$$ (9)

This formula is a result of Eq (7). Eq (9) calculates the instantaneous movement of the tumor particle and ensures that the motion of particle with respect to the length of cylinder is normal to its smooth motion curve in the circular cross-section.

Here, I show that Eq (9) is rational using the least-action principle. When examining the instantaneous behavior of the particle, Δ*s* and Δ*t* are close to zero. approach zero. According to the definition of an infinitesimal, when two or more infinitesimals simultaneously approach zero, they may be compared by considering their ratio [18]. Thus, we can use the following approximation:

$$a=\frac{d^{2}s}{dt^{2}}=c$$ (10)

where *c* is a constant. Because *v*_0_ is much smaller than the velocity of light, Newton’s second law states that the mass of the tumor particle is constant. We can conclude that the force on the particle is always constant in an instantaneous state, and so we obtain

dw=F·ds (11)

where *dw* is the work done in carrying the tumor particle from one point to another in an instantaneous state and *ds* is the differential vector displacement along the path.

Because the work depends only on *ds*, its magnitude is determined by the length of the path. We apply this fact in the following way. Let us choose *p*_0_ as a starting point and *p*_1_ as an ending point. Let *p*_1_ be within a plane of the circular cross-section, and let the distance between *p*_0_ and the plane be close to zero; we call this distance *dD*. When *θ* is the angle between *ds* and *r*, we have

$$ds=\frac{dD}{sin\theta }$$ (12)

This equation indicates that due to the first derivative theorem for local extreme values [17] and because *sinθ* = *cos*′ *θ*, *ds* has a local minimum value when *θ* is equal to *π*, Because the work depends only on *ds*, *dw* has a local minimum value when *θ* is equal to *π*.

As discussed previously, in the least-action principle for tumor particles, a particle investigates all of the paths in the neighborhood and then chooses the path with the least action. Thus, it is easy to understand that the optimal path must have a minimum value. In other words, as mentioned previously, a tumor particle will not choose a path unless *dw* is minimized.

In short, we must satisfy a certain condition, i.e., *ds* must be instantaneously normal to the curve of *r*. For a particle moving along the length of the cylinder to reach its destination in the shortest time, the motion of the particle must be vertical with respect to the circular cross-section.

**Fig 2:**
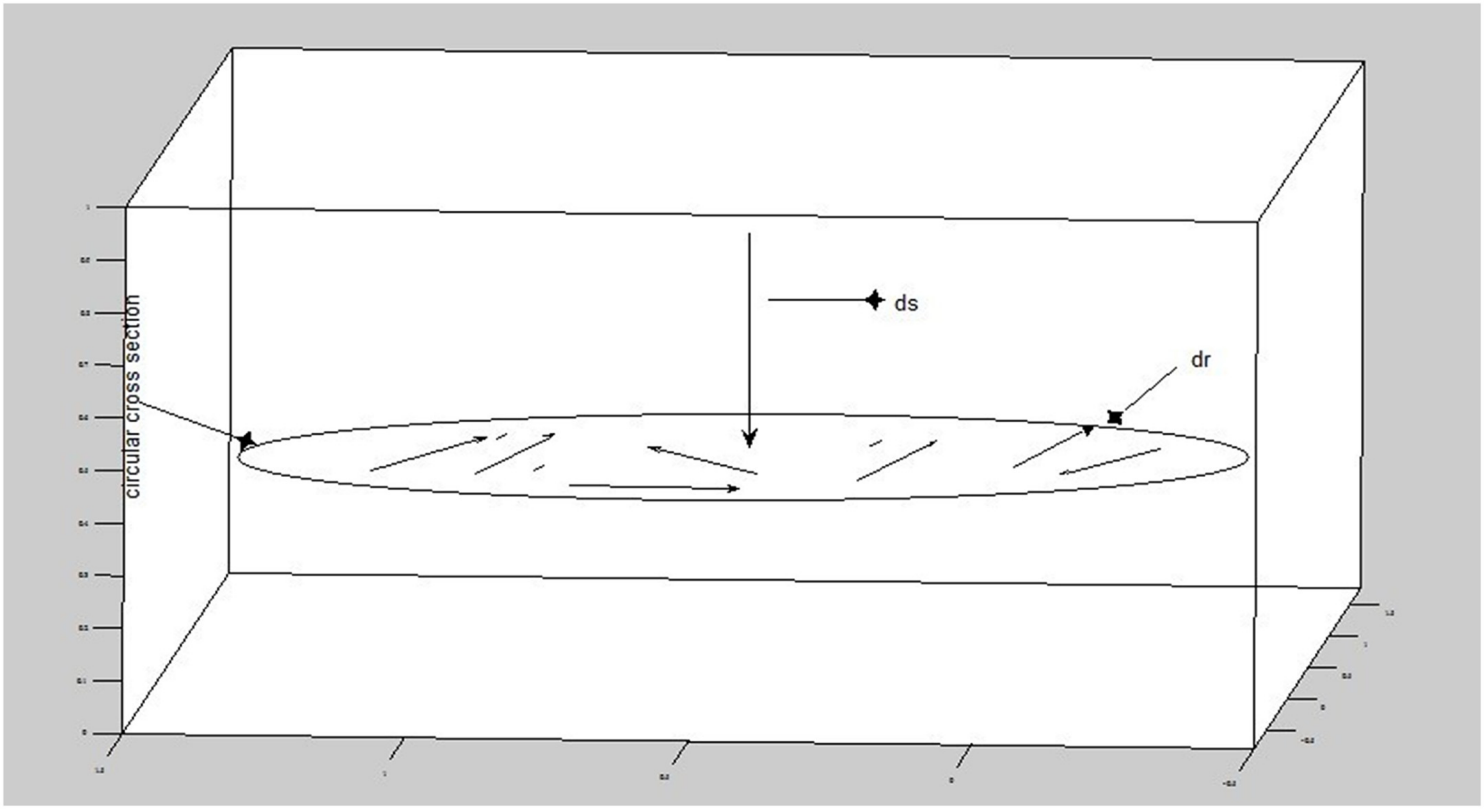
The optimal path of the tumor particle must be instantaneously normal to an arbitrary curve in the circular cross-section.

An important property of the particle’s movement arises, which we will use in our description of the principle of least action. We called this property randomness. Suppose there are countless curves along two types of paths: the length of the cylinder and the circular cross-section. Because *ds* is instantaneously normal to the curve of *r* and the curve of *r* is randomly determined in the circular cross-section, we find the right path if and only if *ds* is perpendicular to all possible curves of *r* and *ds* is unique(Fig 2):

$$p_{right}\Leftrightarrow (\exists !ds)(\forall r)(ds⊥r)$$ (13)

*p*_*right*_ is the path for which the action is minimized.

However, Eq (13) is not sufficient to determine why hepatic metastasis is not dependent on the proximity to its primary origin after entering the blood-flow pathway. Thus, we supplement our model with another aspect of flow: viscosity. To determine the fluid state in a portal vein system, the Reynolds number must be measured. The Reynolds number is the most important dimensionless number in fluid mechanics. This parameter is used to determine whether a flow is laminar, turbulent or in transition. For this model, we must know the material parameters for each part of the portal vein. In 2011, C C Botar et al. analyzed the fluid dynamics in a portal vein system [19].

Under normal conditions, the Reynolds number reaches a maximum value in the main portal vein. In cases of cirrhosis, the blood rheology of the portal vein changes. One common effect is a decrease in blood viscosity [20]. In other words, the value of Re in cirrhosis is lower compared to normal conditions. Nevertheless, the actual parameters are not the focus of our model; our primary focus is the distribution of Re.

It is well known that the Reynolds number in a pipe flow is determined by the diameter [21]. The larger the blood vessel diameter, the larger the Reynolds number. Thus, it is clear that the Re of the main portal vein is a maximum for the maximum blood vessel diameter in the portal vein system, which has been observed in live cirrhosis cases.

In this model, based on previous data [19] and analysis results [20], the range of Re is approximately 1020–2600 in the main portal vein, which falls into the transition and laminar region (transition flow:2300 < Re < 4000, turbulent flow: Re ≥ 4000). We note that eddies can exist in a laminar flow, not just in turbulent or transition flows. Vortices arise for Re values of approximately 40 or higher [10]. As Re increases, the frequency and duration of the turbulence also increases until > 4000, at which point the turbulence is persistent.

These turbulent eddies create fluctuations in velocity and are one of the driving forces of tumor particles. In this situation, we do not need to alter our previous logical analysis. On the contrary, we will now use a mathematical technique to separate the flow velocity using Eq (14). This technique is commonly called Reynolds decomposition [22]. We decompose the flow as follows:

$$\begin{array}{ccc} r(t) & = & r+r^{\prime }(t)\\ s(t) & = & s+s^{\prime }(t) \end{array}$$ (14)

As shown in Fig 2, *r*(*t*) is the actual motion in the circular cross-section, *r* is the actual motion under idealized conditions, and *r*′(*t*) represents turbulent fluctuations. The second equation is the same as the first, corresponding to the longitudinal direction.

The turbulent motions associated with eddies are approximately random. When applying Reynolds decomposition, it is straightforward to introduce the random walk hypothesis [23]. We illustrate this hypothesis for the circular cross-section. Suppose we have a tumor particle A in an ideal fluid; in this case, the particle can move in any direction step by step, where the distance of each step is one unit, which is constant. We write $p=\frac{1}{360k}$(*kϵR* ^+^ and *k* follow a reciprocal relationship with respect to the degree of each step’s arc) for the probability of A’s movement in all directions. In addition, let 1 be the distance for one unit and let the time interval for each step be infinitesima(every step is determined by equal intervals of time, but not distance. owing to time interval at instantaneous state, the distance of each step is equal in the same circular cross section). If all of the steps for A that occur in disjoint time intervals are independent, i.e., if the particle motion is independent in the *n*_*th*_ time interval of step *n*, the probabilistic model can be described as follows:

$$p\;(X_{1})=\frac{1}{360k}$$ (15)

$$p\;(X_{n})={\left(\frac{1}{360k}\right)}^{n}$$ (16)

where *X*_*n*_ is the location of the tumor particle at step *n* and (*X*_*n*_:*nϵN*) is a group of independent random numbers. Now we write *W*_0_ for the original position of the tumor particle, and its location for the *n*_*th*_ time interval is

$$W_{0}=W_{0}+X_{1}+X_{2}\ldots ..+X_{n}$$ (17)

The set of these random variables is (*W*_*n*_:*n* = 0,1,2…)), which we refer to as “a random walk”. The sample size Ω of (*W*_*n*_) is

$$\Omega =[\omega :\omega =(\omega_{0},\omega_{1},\omega_{2}\ldots ),\omega_{i}\epsilon Z]$$ (18)

Then, we obtain

$$W_{n}\;(\omega )=\omega_{n}$$ (19)

Now we can examine the viscosity to improve the model for a “random walk” using the Reynolds number.

Suppose that A accelerates in response to turbulent fluctuations in the Reynolds numbers in the circular cross-section. In this case, following the same path described above, A will arrive at *W*_*n*_ in the (*n* − *m*)_*th*_ step (*m* < *n*), and the distance for this case is one unit greater than in the previous ideal fluid. Then, we have

$$W_{0}=W_{0}+X_{1}+X_{2}\ldots ..+X_{n-m}$$ (20)

Thus, we obtain a new probabilistic equation for step (*n* − *m*):

$$p\;(X_{n-m})={\left(\frac{1}{360k}\right)}^{n-m}$$ (21)

Using the definition of an exponential function, we conclude that:

$$p\;(X_{n})>p\;(X_{n-m})$$ (22)

Now we consider the collision probability of the tumor particles (including collisions with other tumor particles and the vessel wall). Suppose *W*_*n*_ is the position for a collision between A and another tumor particle (or the vessel wall). To use our probabilistic model with such a description, we replace the initial definition of *p* by the probability of the instantaneous collision between A and another tumor particle (or the vessel wall). Thus, from Eq (22), it is clear that the collision probability is close to the value of Re. Moreover, this result indicates that these particles traveling through the portal vein system tend to collide with each other in the main portal vein if tumor particles are uniformly distributed throughout the whole system. However, in reality, this is not the case. A Japanese study reported that PVTT does not have a preference for evading any part of the portal vein system [2]. Now, we consider the concentration *ρ* of the tumor particles to further develop our model. A change in concentration *ρ* can arise from a difference in the location of the primary tumor. Thus, we must consider how the PVTT model is affected when *ρ* is incorporated.

We define *ρ*_*m*_ as a minimum and uniform distribution density for the formation of a tumor thrombus in the portal vein system. Suppose we measure the instantaneous density of a circular cross-section and denote this value as *ρ*_*i*_. *ρ*_*i*_ in our model can be considered a parameter. Obviously, if *ρ*_*i*_ changes, the location of the PVTT changes. Can we qualitatively describe this change in the PVTT location? The sign of *ρ*_*i*_ is crucial. To take this restriction into account, we consider two cases for our model:

1. When *ρ*_*i*_ < *ρ*_*m*_, it is straightforward to verify that there is no portal vein tumor thrombus (regardless of whether Re is included).
2. When *ρ*_*i*_ exceeds *ρ*_*m*_, we encounter the problem of particle collision under the impact of Re. Here, we focus on the model of a “Random walk” again.

We first consider collisions when *ρ*_*i*_ = *ρ*_*m*_. Under this condition, for convenience, we define *W*_*n*_ as the location of the particle collision (*W*_*n*_ is the same for any circular cross-section). Let *p*_0_ be the distance of particle A from *W*_*n*_ in any circular cross-section, and let *W*_0_ be the initial site in this instantaneous state. Suppose that particle A “takes a walk” along the same path until a collision occurs at *W*_*n*_.

From our derivation, it is clear that the collision event is determined by two factors, the Reynolds number and *ρ*_*i*_. The change in *p* arises from the change in *ρ*_*i*_ ((*p* is the distance of A from *W*_*n*_ when *ρ*_*i*_ is changed), as described by the following formula:

$$\rho >\rho_{i}\Rightarrow p<p_{0}$$ (23)

Let us suppose that *W*_1_ is the initial site for *p* is changed. The location of *p* is changed. the location of *W*_*n*_ for the *i*_*th*_ time interval is

$$W_{n}=W_{1}+X_{1}+X_{2}\ldots ..+X_{i}$$ (24)

Thus, we have a probabilistic equation for step *i*:

$$p\;(X_{i})={\left(\frac{1}{360k}\right)}^{i}$$ (25)

We now take the main portal vein in this instantaneous state and find its probabilistic equation. Let *W*_*s*_ be the starting point, *p*_*m*_ be the distance from *W*_*s*_ to *W*_*n*_, and *s* be the number of steps taken by particle A to travel the distance *p*_*m*_. In addition, each step in *s* and *i* corresponds to an equivalent time interval. The probabilistic equation is

$$p\;(X_{s})={\left(\frac{1}{360k}\right)}^{s}$$ (26)

1. When *s* < *i*, particle collisions tend to occur in the main portal vein.
2. When *s* > *i*, collisions do not tend to occur in the main portal vein.

When considering fluctuations in both Re and *ρ*_*i*_, *i* for the circular cross-section reaches the minimum number of steps in this state. Thus, the collisions tend to occur in the circular cross-section.

Can we answer our original question with the current model? The answer is again no. Our model is an instantaneous model. The formation of a tumor thrombus is determined by numerous instantaneous states of our model. Thus, this circular cross-section must be analyzed in a four-dimensional space, denoted as *R* ^4^.

Let *T*_*n*_ be an instantaneous state during the formation of the tumor thrombus and $R_{n}^{3}$ be the three dimensional space at *T*_*n*_. Now we define *R* ^4^ to be the set of all lists of *R* ^3^ during the formation of the tumor thrombus:

$$R^{4}=[R^{3}:\;(R_{1}^{3},R_{2}^{3},R_{3}^{3}...R_{n}^{3})]$$ (27)

We had already shown that the probabilistic Eq (26) corresponds to the collision event in an instantaneous state before we considered a “4D” case. For four-dimensional space

$$p\;(R^{4})=\prod_{l=1}^{n}p\;(R_{l}^{3})=\prod_{m=1}^{n}{\left(\frac{1}{360k}\right)}^{i(m)}$$ (28)

where *p*(*R* ^4^) is the probability of a collision in a four-dimensional space and $p\left(R_{l}^{3}\right)$ is the probability of a collision in an instantaneous state.

Here, we should note an important fact. Eq (28) describes the majority of the collision events, but does not consider the particles’longitudinal motion. The case for longitudinal displacement depends only on the fact that *W*_*n*_ must coincide with the initial site. In the case of linear motion, there are only two alternatives: the collision event occurs in a single direction or in both directions. In other words, there are two values for a collision event’s probability:$\frac{1}{2}$ and 1. Now, let us suppose that L is the time point for these events and that the probability is $\frac{1}{2}$. We modify Eq (28) as follows:

$$p\;(R^{4})={\left(\frac{1}{2}\right)}^{L}.\prod_{m=1}^{n}{\left(\frac{1}{360k}\right)}^{i(m)}$$ (29)

In addition, the *ρ* and Re values for adjacent circular cross-sections should be approximately equal. After ensuring that the minimum product of the probability of step *i*(*m*) for all *R* ^3^ and ${\left(\frac{1}{2}\right)}^{L}$ is met, it is clear that the tumor thrombus tends to locate at the position of the portal vein containing a minimum *p*(*R* ^4^) in a circular cross-section.

## Discussion

### Mass force, Womersley number, and flexibility of the vessel wall

I have demonstrated that this model can answer our question about tumor thrombus: why are PVTT’s common in the major branch of the portal vein? However, this is not quite true because the blood stream is governed by other influential factors such as the mass, Womersley number, and flexibility of the vessel wall.

Now, we must consider how the PVTT model is affected when these factors are incorporated. We begin by considering gravity. In Eq (10), t approaches zero. The fluid of the model, therefore, approximately corresponds to the theory of fluid at rest because there is only one stress-pressure in a static fluid [10]. For a quiescent state, the mass force per unit volume added to the stress-pressure force per unit volume must give zero [10]:

-Δp-ρΔϕ=0 (30)

where Δ*p* is the pressure force per unit volume and Δ*ϕ* is the potential energy per unit mass.

In general, the density *ρ* varies throughout the portal vein system in an arbitrary way, but for the portal vein system as a whole, the fluid of our model tends to remain static in the instantaneous state. Thus, it is reasonable that the pressure must balance gravity to reach equilibrium.

Let us now consider the Womersley number. In general, this number is based on the pressure gradient and flow velocity in an arterial pulse, where the blood flow and pressure are unsteady [24]. However, according to the definition of damping, when these pulsations are far from the heart, they are damped in small vessels such as capillaries and veins. When *α* is below 10, the flow can be approximated as a steady flow [25]. Christophe Van Steenkiste et al. measured the Womersley number in the portal vein for rat models of portal hypertension and cirrhosis [26] and found it to be small, indicating a quasistatic approximation. Moreover, the Womersley number also affects the local hemodynamic wall parameters. For a minuscule value of *α*, it is valid to consider a rigid wall and a steady flow in our portal vein system.

### Relationship between the tumor microenvironment and the probability of collision events

Several studies have shown that microenvironmental changes can influence the development of neoplasia [27, 28, 29, 30]. These data reveal that perturbations in the environment of a cell can alter the signal provided to the parenchymal cells of a tissue. These altered parenchymal cell signals can result in neoplasia. Other models have indicated that the interface between a niche and its occupants can select for specific characteristics [31], although a competitive selection process has not yet been independently verified. Further studies [32] have reported that stem cell-niche interactions change the competitive dynamics of a cell population within a tissue. Specifically, the likelihood that niche cells will acquire genotypic changes that introduce variability into the niche and affect the conditions for parenchymal cells is, in part, dependent on the dynamics of the niche cell population [27].

An important characteristic of dynamic change is a two-way conversation between niche cell populations. From an evolutionary point of view, normal and malignant cells competing for nutrition cannot coexist in a tissue. If one of these two cell populations is superior in numbers, they will form a full-support ecologic niche via complex cell signaling and molecular mechanisms. One population will overcome the other, leading to a process from quantitative changes to qualitative changes for niche variability. The result of the tissue’s fate is either malignant tumor or normal tissue.

Physically speaking, in our model, if the collision probability for the tumor particles (including collisions between particles and the blood vessel wall) is too low, the particles would not be expected to create a major “field”, even if these particles include stem tumor cells and niche tumor cells. The potential for an acquired change, causing particles to combine and develop complementary changes in parenchymal cells to result in neoplasia, would be low. In other words, if *R* ^4^ reaches a maximum, mutations could accumulate in a pool of these “outsiders”, creating a field defect. By forming an abnormal field rather than a few tumor particles, the potential for complementary abnormalities allowing for the establishment of abnormal parenchymal cells increases.

Some previous research supports these findings. For example, a study by Dongsu Park et al. [33] revealed that the turnover kinetics of these cells is strikingly high and that the cells are replenished by a stem/progenitor population. Furthermore, the translocation of these cells indicated a more systemic effect on mesenchymal cells from a highly locally constrained pool [32].

In our model, as the collision probability increases, the field effect of the portal vein also increases, resulting in complementary changes in the parenchymal cells. These changes can accumulate and lead to specific niches for a tumor microenvironment. Thus, it is apparent that the collision probability has a significant impact on tumor thrombus formation in any portion of the portal vein.

## Conclusions

In conclusion, the collision probability model presented herein is meaningful for portal vein tumor thrombus formation. For venous metastases of hepatocelluar carcinoma, our model partly explains why researchers have not observed significant differences in the distribution of portal vein tumor thrombus and suggests that an increased collision probability can alter the tumor microenvironment by increasing the number of tumor cells. Furthermore, we have demonstrated how the simple concepts of the “Principle of least action”, “Directional Derivatives”, “Reynolds’ decomposition”, “Random walk”and “probability”can be used to explore the formation mechanism of PVTT, establishing a mathematical model that captures some of the features of cancer metastasis through blood circulation.
