# Correction: A Collision Probability Model of Portal Vein Tumor Thrombus Formation in Hepatocellular Carcinoma

**DOI:** 10.1371/journal.pone.0138165

**Published:** 2015-09-09

**Authors:** Fei Xiong

In the results section, there are errors in Eqs 17, 20, and 22. Please view the complete, correct equations here.

$$W_{n}=W_{0}+X_{1}+X_{2}.....+X_{n}$$ (17)

$$W_{n}=W_{0}+X_{1}+X_{2}.....+X_{n-m}$$ (20)

$$p(X_{n})<p(X_{n-m})$$ (22)
